# Spatio-Temporal Expression Patterns of Connexins 43 and 30 in Hippocampal Astrocytes During Postnatal Development

**DOI:** 10.3390/ijms27188042

**Published:** 2026-09-09

**Authors:** Alejandro Uribe-Arias, Jérôme Ribot, Pascal Ezan, Philippe Mailly, Nathalie Rouach

**Affiliations:** Center for Interdisciplinary Research in Biology, Collège de France, CNRS, INSERM, Université PSL, PSL-Neuro, 75005 Paris, France; alejandro.uribe@unil.ch (A.U.-A.); jerome.ribot@college-de-france.fr (J.R.); pascal.ezan@college-de-france.fr (P.E.); philippe.mailly@college-de-france.fr (P.M.)

**Keywords:** connexin, astrocyte, super-resolution imaging, development

## Abstract

Astrocytes are extensively interconnected via gap junction channels formed primarily by connexin 43 (Cx43) and connexin 30 (Cx30), two proteins that play central roles in intercellular signaling and homeostatic regulation in the brain. Although the functional properties of these connexins have been widely investigated, their spatial organization within astrocytes and its evolution during postnatal development remain poorly characterized. Here, we combined confocal and stimulated emission depletion (STED) super-resolution microscopy to examine the expression, distribution and colocalization of Cx43 and Cx30 immunoreactive puncta in hippocampal astrocytes from postnatal day 15 to adulthood. Quantitative analyses revealed a progressive increase in the number of both Cx43 and Cx30 puncta during development, whereas puncta size remained largely unchanged. Although connexin puncta appeared more distally distributed in mature astrocytes, this shift was fully accounted for by the growth of astrocytes during development. In addition, colocalization between Cx43 and Cx30 increased during maturation, reaching a stable level after postnatal day 30. Finally, STED super-resolution imaging revealed a diversity of connexin arrangements, including isolated puncta as well as complex assemblies composed of multiple neighboring connexin clusters. Together, these findings provide a quantitative characterization of the developmental remodeling of astroglial connexins and identify structural features that may contribute to the maturation of astrocytic networks.

## 1. Introduction

Astrocytes, the most numerous glial cell type of the central nervous system, are now viewed as important contributors to most brain functions, such as processing of sensory information, locomotion or cognition. Their regulatory functions rely on their tight structural and functional interactions with neurons via various mechanisms ranging from regulation of extracellular homeostasis to active signaling [1].

Besides their interactions with neurons, astrocytes display several typical features. At the single-cell level, astrocytes have a complex morphology characterized by numerous fine perisynaptic processes, which occupy a specific non-overlapping territory. Despite limited structural contacts resulting from their individual domain, astrocytes are nevertheless organized in networks due to their extensive connection via gap junction channels. These channels are permeable to molecules with a molecular weight of up to 1.5 kDa, such as ions, energy metabolites, neurotransmitters or second messengers, and provide the structural basis for long-range intercellular signaling. These abundant channels indeed provide direct intercellular communication to astrocytes as they directly connect their cytoplasm via apposition of two hemichannels, or connexons, between cells. Hemichannels are formed by six connexin (Cx) subunits, which are organized in homomeric or heteromeric arrays, and each of which contains four transmembrane domains. Cx form a family of proteins, expressed in almost all cell types, and comprise 21 members in humans and 20 in mice. Astrocytes express primarily two connexins, Cx43 and Cx30, named according to their molecular weight. Astroglial Cxs are thought to play active roles in brain functions in part via intercellular signaling. Such signaling is indeed implicated in many physiological or pathological processes, including intercellular calcium or sodium waves, potassium buffering or neurometabolic coupling [2]. It is noteworthy that mutations in Cx genes can result in functional alterations and diseases, such as oculodentodigital dysplasia (Cx43) or the Clouston syndrome (Cx30). In addition, recent data indicate that the function of Cx actually extends beyond the formation of gap junction channels and intercellular communication and also includes the formation of hemichannels, which can mediate direct exchange with the extracellular space, as well as non-channel functions, consisting of protein interactions, cell adhesion or intracellular signaling [3,4].

Remarkably, Cx43 and Cx30 differ in many aspects: their C-terminal domain, known to be crucial for protein interactions; the biophysical properties [5] of the channel they form and their permeability to molecules; their regulation by neuronal activity [6] and phosphorylation; and their contribution to behavior [7,8,9]. Despite the specific properties of Cx43 and Cx30 and their important role in brain functions, how both Cxs are distributed within an astrocyte and how such spatial distribution changes with development remain elusive. To investigate this, we performed confocal and super-resolution imaging of hippocampal astrocytes in situ during development. We reveal changes in the abundance, distribution and co-localization of astroglial Cx puncta during development, as well as specific Cx puncta patterns in adults.

## 2. Results

### 2.1. Abundance of Cx30 and Cx43 Puncta Within Individual Astrocytes During Postnatal Development

**Figure 1:**
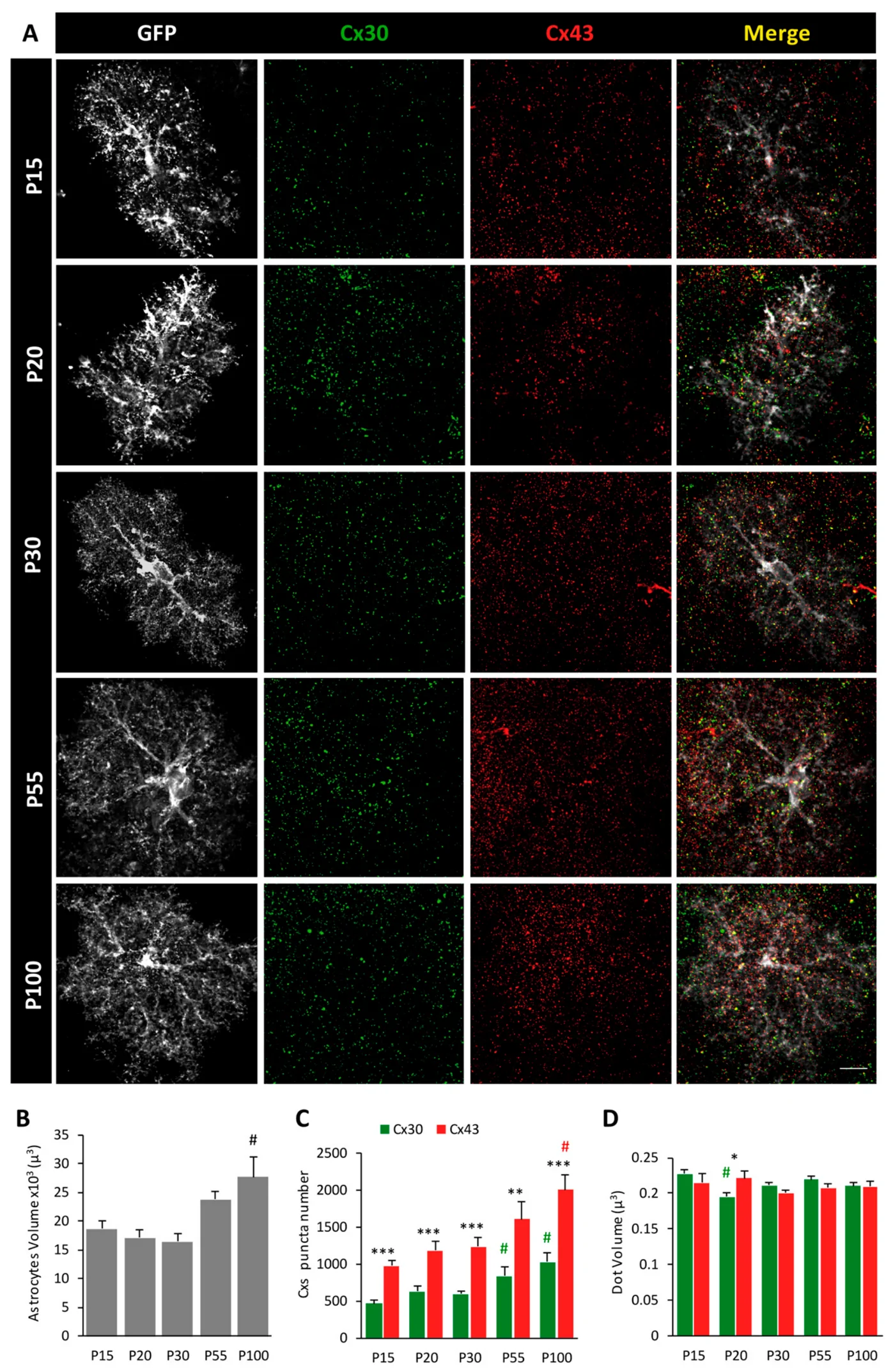
Developmental changes in astroglial Cx30 and Cx43 puncta abundance. (**A**) Representative confocal images of Cx30 (green) and Cx43 (red) immunostaining in individual GFP-labeled astrocytes (white) from GFAP-eGFP mice in the hippocampal CA1 region. Yellow indicates merged Cx30 and Cx43 signals. Representative confocal images at postnatal days P15, P20, P30, P55 and P100. Scale bar: 5 μm. (**B**) Quantification of astrocyte volume during postnatal development. (**C**) Quantification of Cx30 and Cx43 puncta numbers per astrocyte. (**D**) Quantification of individual Cx30 and Cx43 puncta volume. Data are presented as mean ± SEM. # indicates significant differences identified by Tukey’s post hoc test following one-way ANOVA. * indicates significant differences between Cx30 and Cx43 at a given developmental stage (Student’s *t*-test). * *p* < 0.05, ** *p* < 0.01, *** *p* < 0.001.

To characterize the abundance of astroglial connexin puncta at the single-cell level during postnatal development, we immunolabeled Cx30 and Cx43 in hippocampal astrocytes from GFAP-eGFP mice, in which GFAP-positive astrocytes are labeled with cytoplasmic eGFP, allowing us to visualize their entire structure, including fine processes. We evaluated expression at postnatal days (P) 15, 20, 30, 55 and 100 and acquired images with confocal imaging (Figure 1A).

We first quantified astrocytic volume and found a significant increase during postnatal development (P15: *n* = 23, P20: *n* = 23, P30: *n* = 24, P55: *n* = 17, P100: *n* = 25; one-way ANOVA, F = 8.28, *p* < 0.0001, Figure 1B). While the volume of astrocytes remained stable throughout the first postnatal month (~17,000 µm^3^, Tukey post hoc test, *p* > 0.9 between P15, P20 and P30), we observed an increase at P55 (23,800 ± 1400 µm^3^) that became significant at P100 (27,700 ± 3300 µm^3^, Tukey post hoc test, *p* < 0.001 compared to P15, P20 and P30).

We next quantified Cx30 and Cx43 puncta within individual astrocytes (Figure 1C). At all examined developmental stages, Cx43 puncta were significantly more abundant than Cx30 puncta (Student’s *t*-test, *p* < 0.05 for each age). Moreover, both connexins displayed a progressive increase in puncta number during postnatal development (one-way ANOVA; F = 6.97, *p* < 0.0001 for Cx30; F = 6.45, *p* < 0.0001 for Cx43). The abundance of both Cx30 and Cx43 puncta reached its highest level at P100 (*p* < 0.01 compared to P15, P20 and P30 for Cx30 and for Cx43; Tukey post hoc test).

Finally, we evaluated changes in the volume of individual connexin puncta (Figure 1D). Cx43 puncta volume remained remarkably stable throughout development (~0.2 µm3; one-way ANOVA; F = 0.81; *p* = 0.81). Similarly, Cx30 puncta volume showed only limited developmental variation despite a significant overall effect (one-way ANOVA, F = 4.68, *p* = 0.0016), with a transient decrease observed at P20 compared with P15 and P55 (Tukey post hoc test). Consistent with this observation, Cx30 puncta were smaller than Cx43 puncta only at P20 (Student’s *t*-test, *p* < 0.01), whereas no differences were detected at other developmental stages.

Together, these results indicate that postnatal astrocyte maturation is accompanied by a progressive increase in astrocytic volume and in the abundance of both Cx30 and Cx43 puncta. In contrast, the size of individual connexin puncta remained largely unchanged throughout development. Notably, Cx43 puncta were consistently more abundant than Cx30 puncta at all examined developmental stages.

### 2.2. Spatial Distribution of Connexins Within Astrocytes

**Figure 2:**
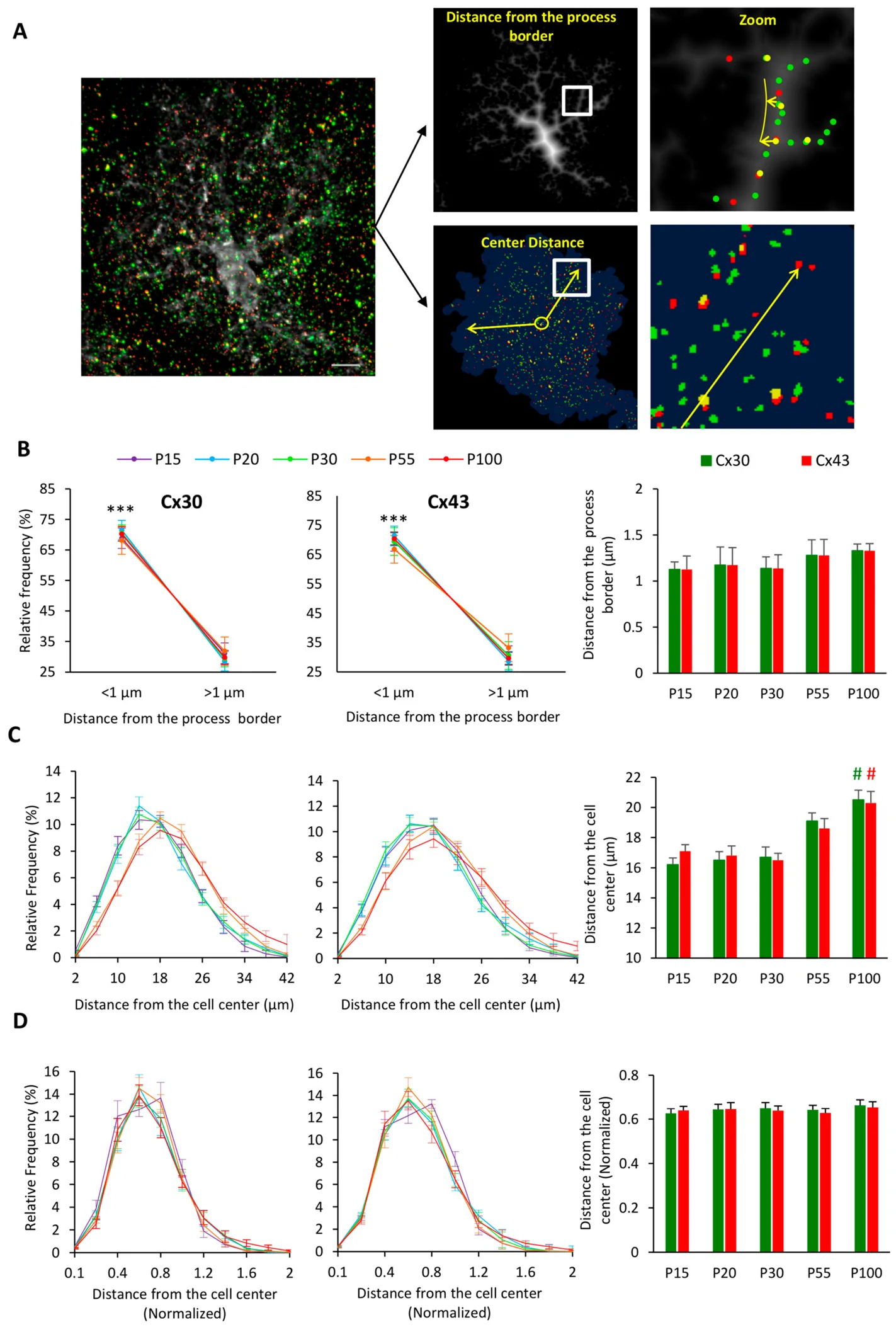
Spatial distribution of connexins within astrocytes during postnatal development. (**A**) Schematic representation of the analyses used to assess connexin spatial distribution within astrocytes. Distances were measured either relative to astrocytic processes (**top right panels**) or to the astrocyte centroid (**bottom right panels**). White boxes indicate the regions shown enlarged in the adjacent zoom panels. Yellow lines/arrows represent the measured distance to the process border or to the cell center. (**B**) Spatial distribution of Cx30 and Cx43 puncta relative to astrocytic processes. (**Left panels**) show the proportion of puncta located within 1 μm of astrocytic process borders. (**Right panel**) shows the mean distance of connexin puncta from astrocytic processes. (**C**) Spatial distribution of Cx30 and Cx43 puncta relative to the astrocyte centroid. (**Left panels**) show the distributions of Cx30 and Cx43 puncta distances from the cell center at different developmental stages. The **right panel** shows the mean distance of connexin puncta from the cell center. (**D**) Same as in (**C**) after normalization for astrocyte size. Centroid-to-punctum distances were normalized by the cube root of the mean astrocyte volume at each developmental stage. Data are presented as mean ± SEM. # indicates significant differences identified by Tukey’s post hoc test following one-way ANOVA, # *p* < 0.05. *** indicates significant differences between Cx30 and Cx43 at each developmental stage (Student’s *t*-test). *** *p* < 0.001.

We next examined the spatial distribution of Cx30 and Cx43 puncta within astrocytes by analyzing their position relative to astrocytic processes and to the cell center (Figure 2A).

We found that the majority of Cx30 and Cx43 puncta (~70%) were located within 1 µm of astroglial processes (Student’s *t*-test, *p* < 0.001 for each developmental stage and each connexin; Figure 2B, left panels). This proportion did not differ between Cx30 and Cx43 (Student’s *t*-test, *p* > 0.05 for each developmental stage), and the proportion of connexins close to processes did not significantly change during development (one-way ANOVA, F = 0.17, *p* = 0.96 for Cx30; F = 0.24, *p* = 0.92). Consistently, the mean distance of Cx43 and Cx30 puncta to the astroglial processes remained close to 1 µm and did not vary during development (one-way ANOVA, F = 0.48, *p* = 0.75 for Cx30; F = 0.06, *p* = 0.99; Figure 2B, right panel). These data indicate that both Cx43 and Cx30 are preferentially localized near astrocytic processes and maintain a remarkably stable distribution throughout postnatal development.

We then investigated the spatial distribution of Cx puncta by analyzing their position relative to the center of astrocytes, defined as the centroid of the astrocyte’s volume (Figure 2C). The spatial distributions suggested that both Cx43 and Cx30 relocated further away from the cell center in adult astrocytes (P55 and P100) than at the juvenile stage (P15, P20 and P30) (Figure 2C, left panels). To quantify this observation, we calculated the mean distance of connexin puncta from the astrocyte centroid (Figure 2C, right panel). We found that for both Cx30 and Cx43, this distance significantly increased by ~30% in P100 adult mice compared to juvenile mice (one-way ANOVA, F = 6.58, *p* < 0.0001 for Cx30; F = 5.24, *p* = 0.0007 for Cx43; Tukey post hoc test, *p* < 0.005 against P15, P20 and P30).

Finally, we asked whether this distal distribution reflected a genuine repositioning of connexin puncta within astrocytes or simply resulted from the developmental expansion of astrocytic territory (Figure 1B). To address this question, we normalized the distance of connexin puncta from the cell center by astrocyte volume. Following normalization, the shift toward larger distances observed at P55 and P100 was no longer apparent in the distributions of either Cx30 or Cx43 puncta (Figure 2D, left panels). Consistent with this observation, the normalized mean distance of both connexins remained unchanged throughout postnatal development (One-way ANOVA, F = 0.33, *p* = 0.85 for Cx30; F = 0.16, *p* = 0.96 for Cx43, Figure 2D, right panel). These findings indicate that the apparent distal redistribution of connexin puncta during maturation largely reflects astrocyte growth rather than a developmental reorganization of connexin positioning.

### 2.3. Colocalization of Astroglial Cx30 and Cx43 During Postnatal Development

**Figure 3:**
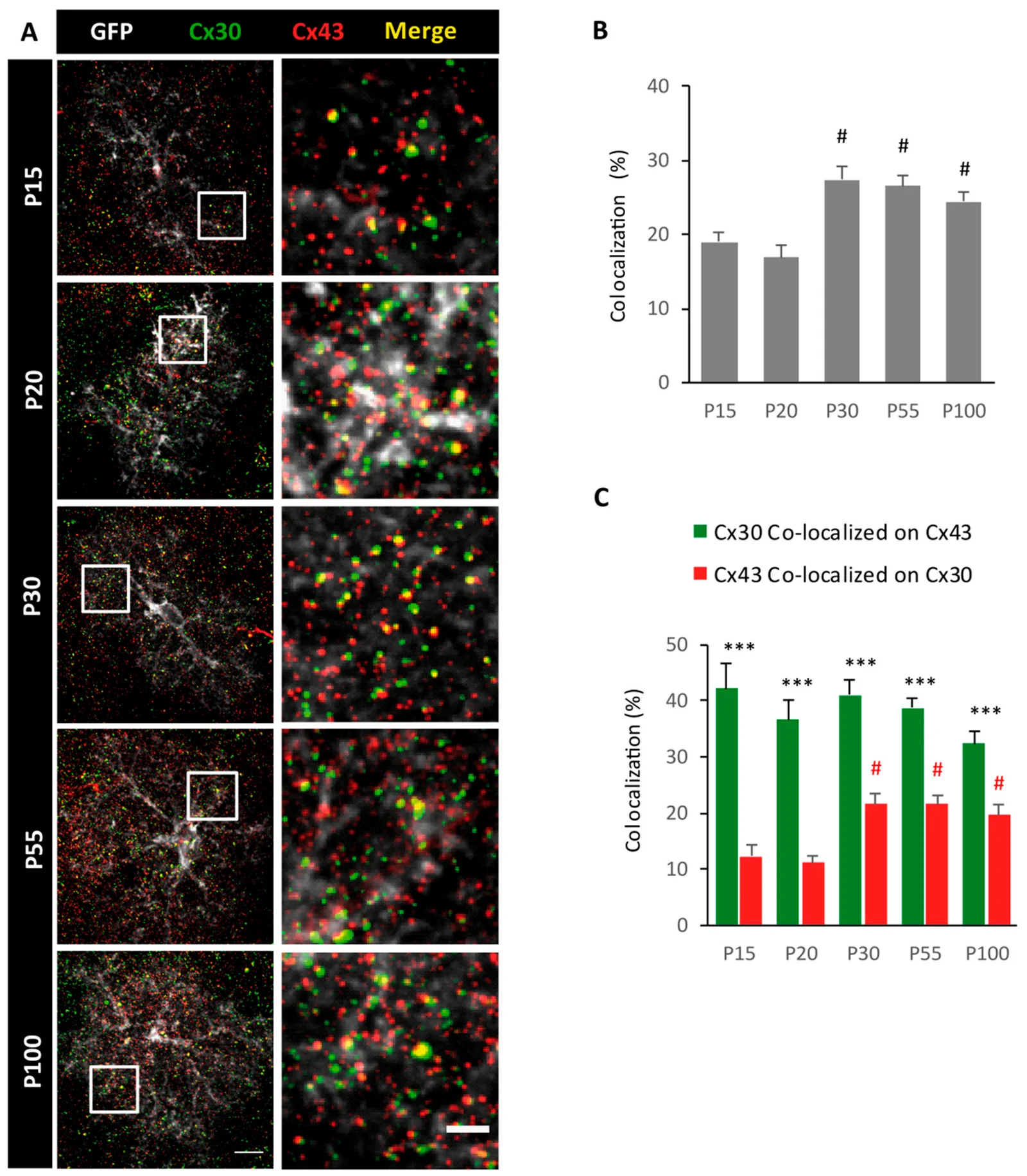
Colocalization of astroglial Cx30 and Cx43 during postnatal development. (**A**) (**Left**) Representative confocal images of GFP-labeled hippocampal CA1 astrocytes (white) immunostained for Cx30 (green) and Cx43 (red). White squares indicate the regions enlarged in the adjacent panels. Scale bar: 5 μm. (**Right**) Enlarged regions of interest. Scale bar: 2 μm. In both panels, yellow indicates merged Cx30 and Cx43 signals. (**B**) Quantification of volume-based colocalization between Cx30 and Cx43 puncta. For each postnatal stage, colocalized puncta were normalized to the total number of connexin puncta (Cx30 + Cx43). (**C**) Quantification of connexin-specific colocalization. Green bars represent the proportion of Cx30 puncta colocalized with Cx43, whereas red bars represent the proportion of Cx43 puncta colocalized with Cx30. Data are presented as mean ± SEM. # indicates significant differences identified by Tukey’s post hoc test following one-way ANOVA. # *p* < 0.05. *** indicates significant differences between Cx30 and Cx43 at a given developmental stage (Student’s *t*-test). *** *p* < 0.001.

We next investigated the colocalization between the two astroglial connexins during postnatal development using a volume-based colocalization analysis (Figure 3; see Section 4.3). Given the spatial resolution of confocal microscopy, this analysis reflects spatial proximity between Cx30 and Cx43 puncta rather than their molecular association within the same gap-junctional assembly. Representative confocal images illustrate Cx30 and Cx43 immunostaining within individual astrocytes across developmental stages (Figure 3A). The enlargement of selected regions of interest revealed discrete puncta of colocalization between Cxs (Figure 3A, yellow punctum, right panel). Quantitative analysis showed that ~20% of the total connexin puncta population was colocalized at P15 and P20 (Figure 3B). This proportion significantly increased during postnatal development (One-way ANOVA, F = 6.59; *p* < 0.0001), reaching ~30% from P30 onwards (Tukey post hoc test, *p* < 0.05 against P15 and P20).

Finally, we investigated the contribution of each connexin to the observed colocalization pattern (Figure 3C). At all developmental stages, a significantly larger proportion of Cx30 puncta colocalized with Cx43 than vice versa (Student’s *t*-test, *p* < 0.0001 for all ages). This asymmetry likely reflects the greater abundance of Cx43 puncta relative to Cx30 throughout development (Figure 1C). While the proportion of colocalized Cx30 remained stable at ~40% across developmental stages (one-way ANOVA, F = 1.85, *p* = 0.13), the proportion of colocalized Cx43 increased significantly during postnatal maturation (one-way ANOVA, F = 10.24, *p* < 0.0001), approximately doubling after P30 compared with P15 and P20 (Tukey post hoc test, *p* < 0.05 against P15 and P20).

### 2.4. Patterns of Connexin Puncta Revealed by STED Super-Resolution Imaging

**Figure 4:**
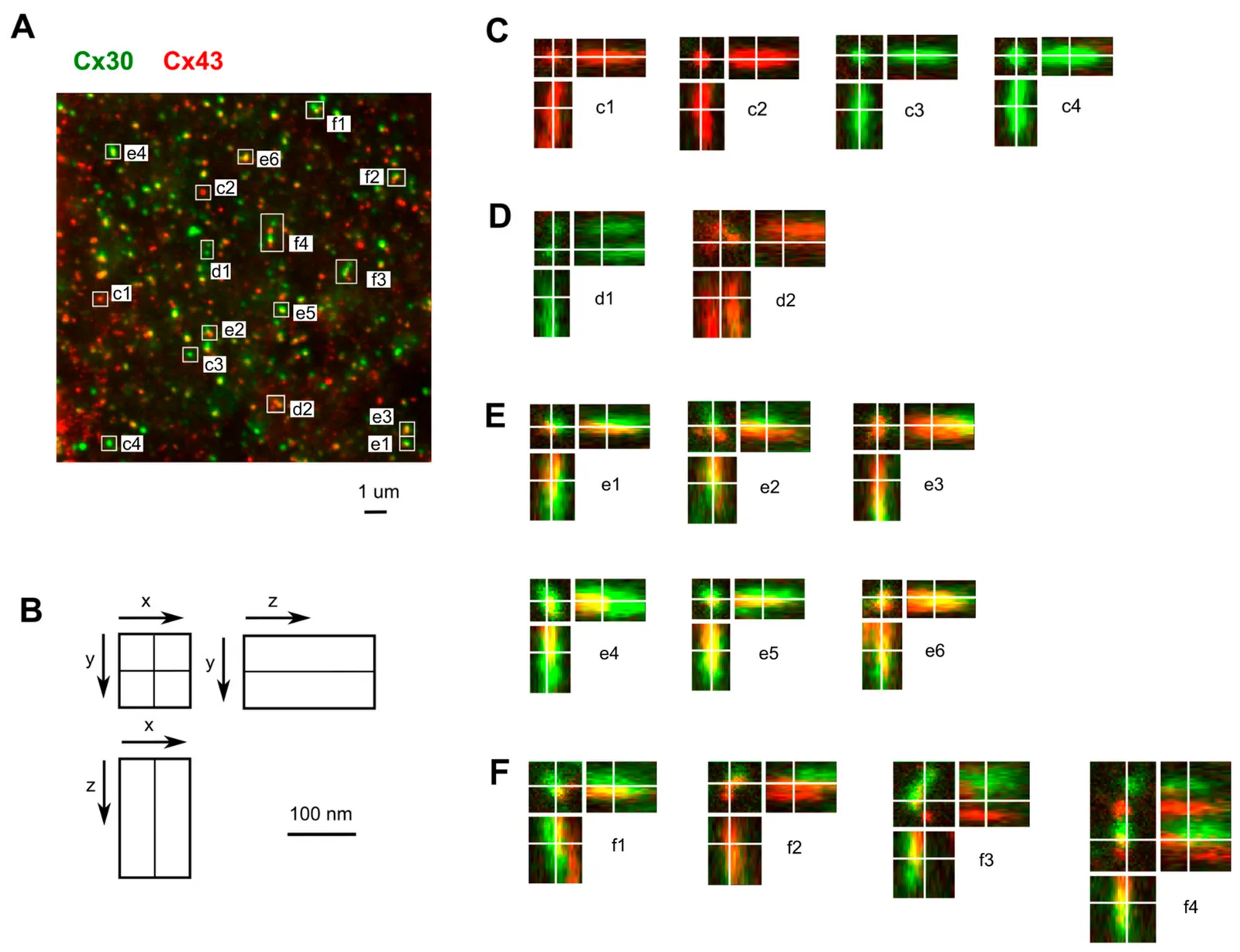
Spatial arrangements of Cx30 and Cx43 revealed by STED super-resolution imaging. (**A**) Z-projection over a 1 µm depth of Cx30 (green) and Cx43 (red) puncta. Images were acquired with 30 nm resolution in x and y. Boxes indicate regions of interest shown at higher magnification in panels (C–F). (**B**) Illustration of the orthogonal-view representation used for the selected regions of interest. (**C**) Examples of individual Cx puncta of variable size: (**c1**,**c2**) Cx30 puncta; (**c3**,**c4**) Cx43 puncta. (**D**) Examples of close associations between puncta of the same connexin type: (**d1**,**d2**) closely associated Cx puncta. (**E**) Examples of doublets formed by adjacent Cx30 and Cx43 puncta: (**e1**–**e6**) heterotypic doublet arrangements. (**F**) Examples of more complex arrangements involving multiple connexin puncta: (**f1**–**f3**) triplet arrangements; (**f4**) quintuplet arrangement.

To visualize the nanoscale organization of astroglial connexins, we performed STED super-resolution imaging in the hippocampal CA1 region at 30 nm spatial resolution in x and y. Figure 4A represents a z-projection of Cx30 and Cx43 immunostaining over a 1 µm depth, while selected regions of interest are displayed in orthogonal view in Figure 4B. STED imaging revealed a diversity of connexin puncta arrangements. Isolated puncta containing either Cx30 or Cx43 were found throughout the sampled regions and displayed variable sizes (Figure 4C; see Figure 4C(c1,c2) for Cx30 and Figure 4C(c3,c4) for Cx43). In addition, closely associated puncta of the same connexin type were observed (Figure 4D(d1,d2)). Associations between Cx30 and Cx43 puncta were also detected, resulting in numerous heterotypic doublet arrangements that could be resolved either in the xy plane or along the z axis (Figure 4E(e1–e6)). Finally, more complex organizations involving multiple neighboring connexin puncta were identified, including triplet (Figure 4F(f1–f3)) and quintuplet (Figure 4F(f4)) arrangements composed of both connexin types (Figure 4F). A visual estimation of these spatial arrangements indicated that single puncta were predominant (~55–65%), while arrangements involving puncta of the same connexin type accounted for ~10–15% and mixed Cx30/Cx43 arrangements for ~20–30% of the observed structures. Together, these observations reveal a diversity of nanoscale spatial organizations of astroglial connexins within hippocampal astrocytes in situ.

## 3. Discussion

In the present study, we quantitatively characterized in mouse hippocampal astrocytes the spatial and temporal expression patterns over postnatal development of the two major connexins, Cx30 and Cx43, from P15 to adulthood. We show that both connexins progressively accumulate during maturation, while preserving a remarkably stable spatial organization relative to astrocyte morphology. We further demonstrate that the spatial association between Cx30 and Cx43 increases during development and display, as revealed by STED microscopy, a previously unappreciated diversity of nanoscale assemblies within individual astrocytes. Together, these findings provide a comprehensive structural description of the developmental organization of astroglial connexins and establish a framework for analyzing whether and how connexin organization contributes to astrocyte maturation and function.

### 3.1. Postnatal Maturation of Astrocytic Connexin Networks

The developmental profile of Cx30 and Cx43 observed here is consistent with previous studies showing that the two major astroglial connexins follow distinct temporal expression patterns: Cx43 is expressed early on during brain development and progressively adopts the punctate distribution characteristic of mature astrocytes, whereas Cx30 appears later, from approximately the second postnatal week onwards, and progressively increases during postnatal maturation [10,11,12,13].

Interestingly, it has been proposed that the progressive maturation of Cx43 does not primarily reflect early brain morphogenesis but rather accompanies the establishment of functional neuronal circuitry and mature astrocytic functions, including metabolic coupling and potassium buffering [10]. On the other hand, the delayed expression of Cx30 coincides with the period of intense astrocyte structural maturation. During this developmental window, astrocytes undergo extensive morphological remodeling while progressively acquiring their mature physiological properties [14,15]. Consistent with this sequence, neurons have been shown to promote astroglial connexin maturation by inducing Cx30 expression while further increasing Cx43 levels, thereby enhancing astrocytic gap-junctional communication [16]. More recently, Cx30 itself has been identified as an active regulator of astrocyte maturation by controlling astrocyte process extension, polarization, actin cytoskeleton remodeling and mechanical properties, indicating that this connexin is not merely a late developmental marker but also contributes directly to astrocyte morphogenesis [15,17].

An important finding of the present study is that the developmental increase in connexin abundance occurred without detectable changes in the size of individual connexin puncta. This observation suggests that connexin assemblies may represent structural units whose size is maintained within a relatively narrow range during development. Consequently, the expansion of the astroglial connexin repertoire would primarily occur through the addition of new assemblies rather than the enlargement of pre-existing ones.

### 3.2. Conservation of Connexin Spatial Organization During Astrocyte Growth

Previous ultrastructural and immunohistochemical studies show that astrocytic gap junctions are not uniformly distributed over the cell surface but are preferentially localized to specialized membrane domains, including fine astrocytic processes, lamellar extensions and perivascular endfeet [18,19,20]. Accordingly, we found that nearly 70% of both Cx30 and Cx43 puncta remained located within 1 μm of astrocytic processes throughout development, supporting the idea that connexin localization is tightly associated with peripheral astrocytic compartments. Interestingly, a substantial fraction of connexin puncta was not directly associated with the major astrocytic processes. This observation likely reflects several non-exclusive factors, including the dynamic trafficking and turnover of connexins [21] and the non-channel functions of astroglial connexins [22,23].

A second important finding is that the relative spatial organization of astroglial connexins is remarkably preserved throughout postnatal maturation despite the substantial expansion of astrocytic territory. Although the mean distance of both Cx30 and Cx43 puncta from the cell center increased during development, this apparent distal redistribution was entirely explained by the increase in astrocyte volume, indicating that the position of connexins scales with astrocyte growth rather than reflecting a major spatial reorganization. These results, therefore, indicate that the mechanisms governing astrocyte morphogenesis are tightly coordinated with those regulating connexin organization. Interestingly, recent studies have identified Cx30 as a regulator of astrocyte process extension, polarization, actin cytoskeleton remodeling and membrane mechanics [15,17]. Preserving the spatial organization of connexins during development may thus contribute to maintaining coordinated structural remodeling throughout the expanding astrocytic territory. It may also preserve the local signaling functions mediated by astroglial connexins as astrocytes mature.

### 3.3. Progressive Association of Cx30 and Cx43 During Postnatal Maturation

Both Cx30 and Cx43 contribute to gap-junctional coupling between astrocytes, but accumulating evidence indicates that these proteins are not functionally redundant. Cx43 is the predominant astroglial connexin, displaying an earlier and broader expression pattern, whereas Cx30 is expressed later, is largely restricted to gray matter, and exhibits a more specialized subcellular distribution [11,16]. Moreover, while Cx43 is thought to provide the principal framework supporting astrocytic intercellular communication [24] and metabolic homeostasis [25,26], Cx30 has emerged as a key regulator of astrocyte polarity, process extension, perisynaptic remodeling and activity-dependent structural plasticity [15,17,27]. This functional specialization likely explains why the two connexins are co-expressed rather than interchangeable. Consistent with this view, simultaneous deletion of Cx30 and Cx43 produces much more severe phenotypes than alteration of either connexin alone, indicating that these proteins combine complementary rather than redundant functions within astrocytic networks [28].

Our analysis further revealed a progressive increase in the spatial association between Cx30 and Cx43 during postnatal development. Importantly, because colocalization was assessed from confocal images and defined by voxel overlap, these measurements should be interpreted as reflecting spatial proximity between Cx30 and Cx43 puncta rather than demonstrating their molecular association within the same gap-junctional assembly. While approximately 20% of connexin puncta were colocalized at P15 and P20, this proportion increased to nearly 30% from P30 onwards, indicating that the degree of spatial association between the two major astroglial connexins is dynamically regulated during maturation. Previous studies have established that Cx30 and Cx43 coexist within astrocytic gap junctions and that Cx30 expression is initiated only after the onset of Cx43 expression during postnatal development [11,12]. In addition, neurons have been shown to coordinately regulate both connexins by further increasing Cx43 expression while inducing Cx30 in subsets of astrocytes during maturation [16]. Although our data do not allow us to determine whether Cx30 is progressively incorporated into pre-existing Cx43-containing assemblies or whether new mixed junctional assemblies are formed during maturation, they support the notion that the spatial association between the two connexins increases as astrocytes mature. Given the complementary developmental regulation and distinct physiological functions of Cx30 and Cx43, this progressive association may provide the structural basis for the complementary functions attributed to these two connexins during postnatal development.

### 3.4. Nanoscale Organization Revealed by STED

Astrocytic connexins have been extensively studied using conventional fluorescence microscopy, but little is known about their nanoscale organization within individual astrocytes. Recent advances in super-resolution microscopy have considerably improved our understanding of astrocyte nanoarchitecture, revealing the organization of fine astrocytic processes, tripartite synapses and Ca^2+^ microdomains beyond the diffraction limit [29,30,31]. However, relatively few studies have specifically investigated the nanoscale organization of astroglial connexins. Using STED microscopy, we observed a remarkable diversity of connexin arrangements, including isolated puncta, closely apposed puncta of the same connexin type, mixed Cx30/Cx43 doublets and more complex multi-connexin assemblies.

Rather than representing uniform membrane structures, connexin assemblies are now recognized as highly dynamic and continuously undergo formation, remodeling and turnover through tightly regulated trafficking and phosphorylation-dependent mechanisms [21,32,33]. Connexin channels are progressively incorporated into growing plaques, whereas older channels are removed through connexosome-mediated internalization, resulting in a constant renewal of junctional structures [21,34]. Within this dynamic framework, the different nanoscale arrangements observed in the present study may represent distinct stages in the dynamic organization of connexin assemblies.

The physiological properties of connexin assemblies depend not only on connexin expression but also on the local organization of connexin assemblies. Glial connexins form indeed structurally distinct classes of intercellular channels, whose molecular composition influences channel conductance, permeability and intercellular communication [35,36,37,38]. Moreover, subcellular redistribution and altered phosphorylation of Cx43 can profoundly affect astrocytic coupling even without major changes in overall connexin expression [39]. In this context, the diversity of connexin organizations revealed here by STED imaging suggests that astroglial connexin assemblies do not represent a homogeneous population but may instead correspond to structurally distinct organizations with potentially different functional properties. Importantly, neither conventional confocal imaging nor STED imaging allows us to determine whether individual connexin puncta correspond to connexins incorporated into gap junctions, unpaired hemichannels, trafficking intermediates, or other connexin-based structures. STED imaging improves the spatial resolution of connexin arrangements but does not by itself resolve their functional or molecular state.

## 4. Materials and Methods

### 4.1. Animals

Experiments were performed on the CA1 hippocampus of wild-type (WT) (C57BL6/J) mice and GFAP-eGFP transgenic mice, in which enhanced green fluorescent protein (eGFP) is expressed under the human glial fibrillary acidic protein (GFAP) promoter. GFAP-eGFP mice were kindly provided by F. Kirchhoff (University of Saarland, Germany) and have been previously characterized [40]. Animals were group-housed under a 12 h light/dark cycle with ad libitum access to food and water. Both male and female mice were used at postnatal days 15, 20, 30, 55 and 100.

### 4.2. Immunohistochemistry

Mice were anesthetized with a lethal dose of Dolethal (150 µL/10 g) and transcardially perfused with phosphate-buffered saline (PBS), followed by 2% paraformaldehyde (PFA). Brains were removed, post-fixed overnight at 4 °C in the same fixative, and cryoprotected in 30% sucrose for 24–48 h. Coronal sections (40 μm) were cut and processed as free-floating sections. For immunohistochemistry, sections were incubated for 2 h in PBS containing 0.2% gelatin and 0.25% Triton X-100 (PGT).

Primary antibodies were diluted in the same solution and applied overnight at 4 °C. After washing sections in PGT, they were incubated with secondary antibodies for 2 h at room temperature. The following primary antibodies were used: anti-Cx30 rabbit polyclonal (1:500, Zymed Laboratories, South San Francisco, CA, USA), anti-Cx43 mouse monoclonal (1:500, BD Biosciences, San Jose, CA, USA) and anti-GFP chicken polyclonal (1:500, Abcam, Cambridge, UK). Secondary antibodies were goat anti-chicken IgG conjugated to Alexa 488 (1:2000), goat anti-mouse IgG conjugated to Alexa 555 (1:2000), and goat anti-rabbit IgG conjugated to Alexa 647 (1:2000). For STED imaging, the dilution of the secondary antibodies was 1:200. After washing in PBS, sections were mounted in Fluoromount or ProLong Gold Antifade reagent and imaged with either spinning-disk confocal or stimulated emission depletion (STED) microscopy.

### 4.3. Confocal Imaging

Images were acquired using a spinning-disk confocal microscope (CSU-X1, Yokogawa Electric Corporation, Musashino, Tokyo, Japan) equipped with a high-resolution camera (Coolsnap HQ2, Photometrics, Tucson, AZ, USA). Z-stacks were sequentially collected using 488, 561 and 642 nm laser lines with a 60X oil-immersion objective at 0.3 μm intervals. Following deconvolution, image analysis was performed using FIJI (2018 Fiji/ImageJ 1.x) software [41,42] together with Bio-Format [43] and mcib3D [44] libraries to quantify the volume of each astrocyte and the distribution of connexins’ puncta and astrocytic processes within.

For each astrocyte, a three-dimensional object was generated from the GFP signal and used to calculate astrocyte volume. The centroid of this volume was defined as the cell center. To analyze connexin distribution, Cx30 and Cx43 images were first filtered using a median filter, and binary images were generated using the Moments thresholding method. Individual connexin puncta were then segmented in three dimensions and the centroid of each connexin was extracted.

The distance from the cell center was calculated using the centroid coordinates of the astrocytes’ volume and each Cx. To account for developmental changes in astrocyte size, centroid-to-punctum distances d_centroid-to-punctum_ were normalized using a characteristic linear dimension derived from astrocyte volume. For each developmental stage, the mean astrocyte volume V_mean_ was calculated, and its cube root was used as the normalization factor d_norm_ = d_centroid-to-punctum_/V_mean_^1/3^. A color map was generated from each plane of the green channel to reproduce the astrocytes’ processes, then the closer point to the border of these processes from the Cx centroid was obtained to quantify the puncta distance to the cell borders. The co-localization between two puncta was defined as puncta sharing a common voxel. For images of astrocytes, the background was first subtracted, and a median filter was applied as well as a threshold (Triangle method). Segmentation was then performed to obtain dots’ population for each astrocyte.

### 4.4. Super-Resolution STED Imaging

Images with super-resolution were taken using a custom upright STED microscope (Abberior Instruments GmbH, Göttingen, Germany). The microscope is based upon a Scientifica microscope body (Slice Scope, Scientifica Ltd., Uckfield, East Sussex, UK) with an Olympus 100X1.4NA ULSAPO objective lens. It comprises a scanner design featuring four mirrors (Quad Scanner, Abberior Instruments, Göttingen, Germany). Excitation lasers at 488 nm, 561 nm, and 640 nm wavelengths are available (Abberior Instruments, pulsed at 40/80 Mhz). One STED laser at 775 nm (MPB-C, pulsed at 40/80 MHz) is available for imaging at 561 nm and 640 nm excitation wavelengths with super-resolution. The conventional laser excitation and STED laser beams are superimposed using a beam splitter (HC BS R785 lambda/10 PV flat, AHF Analysetechnik, Tübingen, Germany). Common excitation power with pulsed excitation ranges from 10 to 20 uW with STED power intensities of up to 200 mW in the focal plane. Cx30 (red) and Cx43 (far red) were acquired with 30 nm xy resolution. Ten different planes separated by 100 nm in depth were recorded. Bleaching correction based on the histogram matching method was performed in FIJI software, and individual regions of interest were drawn manually and represented in orthogonal view.

### 4.5. Statistical Analysis

All data are expressed as mean ± standard error of the mean (SEM), *n* refers to the number of cells analyzed per experiment. Cells were obtained from 3 animals at P15 (10, 8, and 5 cells per animal), 3 animals at P20 (7, 7, and 9 cells), 4 animals at P30 (5, 6, 9, and 4 cells), 3 animals at P55 (7, 3, and 7 cells), and 3 animals at P100 (10, 4, and 11 cells). All statistical analyses were performed with GraphPad Prism 7.05 (GraphPad sofware, San Diego, CA, USA). Statistical significance between two groups (Cx30 and Cx43 at specific postnatal stages) was evaluated with a two-tailed Student’s *t*-test (indicated in figures with the * symbol). Statistical significance for within-group comparisons (Cx changes during development) was determined with a one-way ANOVA followed by Tukey’s test for post hoc analysis (indicated in figures with the # symbol). Asterisks indicate statistical significance (* *p* < 0.05, ** *p* < 0.01, *** *p* < 0.001).

## 5. Conclusions

Together, our findings provide a structural framework for understanding how astroglial connexins mature during postnatal development. The progressive increase in connexin abundance, the preservation of their spatial organization despite astrocyte growth, and the increased association between Cx30 and Cx43 all support the idea that astroglial connexin assemblies undergo coordinated remodeling rather than simple connexin accumulation. Such coordinated maturation is likely to contribute to the establishment of the homeostatic functions of astrocytes, including intercellular metabolic coupling, potassium buffering and the spatial redistribution of signaling molecules, which progressively emerge during postnatal brain development [16,20].

More broadly, increasing evidence indicates that astrocyte maturation is an essential component of neural circuit development. Beyond providing structural support, developing astrocytes actively regulate synapse formation, maturation and refinement, thereby contributing to the establishment of functional neuronal networks [45,46,47,48]. In this context, our study provides a quantitative description of the developmental organization of the two principal astroglial connexins, offering new insight into how astroglial connexin assemblies are organized and remodeled during normal brain development.

## Data Availability

The original contributions presented in this study are included in the article. Further inquiries can be directed to the corresponding author.
